# Supplementary material for: Over the counter use of topical corticosteroid for skin conditions among patients before attending skin specialist clinic in Nepal: A qualitative study
Source: PLOS Glob Public Health. 2025 Jun 24;5(6):e0004812. doi: 10.1371/journal.pgph.0004812 (PMC12186941; doi:10.1371/journal.pgph.0004812)
Supplement: S2_Text — (DOCX) [file pgph.0004812.s002.docx]

| **Section-I: Socio-demographics** | |
| --- | --- |
| Participant Unique ID |  |
| Date of interview |  |
| Age |  |
| Sex |  |
| Address |  |
| Education |  |
| Profession |  |
| Sign and symptoms |  |
| Diagnosis |  |

| **Section-II: Themes and interviews** |
| --- |

Before discussing the particular conditions, start with warm-up questions.

How has your journey been to the clinic.

Have you been here before?

| **Theme-I: Common skin condition patients experience and normative health seeking behavior** |
| --- |

Let me start by asking your skin problems, what are common skin problem you experience?

Do you have local names for the condition?

What do you do when you have these conditions?

Have you seen your family/ neighbour/ friends having such condition?

What do they do when they have skin conditions?

| **Theme-II:** Current skin condition and health seeking behavior |
| --- |

What is this condition? Is there local name for it?

Why do you think this condition has arisen?

Are there local/herbal medications available for this condition?

Did you seek local/herbal treatment before coming here?

Did you go to local drug store for treatment first? What did He/ She say about this condition and what did He/She do to treat this condition?

Did you get the medicine over the counter (OTC)?

Do you think OTC medication had improved your condition in past and at present?

Why do you seek OTC medication for this condition?

Do other people in your community seek OTC medication for such condition?

Did you seek consultation with doctor? Why didn’t you consulted doctor before for this condition?

Do you think using this drug has done any harm to you?

Were you advised any drug by other person like family and relative?

How much time is needed to visit nearest dermatologist? How costly is it?

| **Theme-III:** Knowledge about the OTC Topical steroid preparation |
| --- |

Do you know what is the composition of this topical you are using? Since how long and how many times a day you are using it? Did you get any instruction on how to use it?

Have you heard about steroid? Do you know what is steroid?

Regardless of composition of medicine, do you think such medicine works?

| **Theme-IV:** Policy related to OTC use of Topical steroid |
| --- |

Do you have any idea about the policies related to the use of topical medicine?

Do you think these medicine needs doctor’s prescription?

Have you been suggested by anyone that these medication should not be used without doctors’ consultation?

**Observation notes:**
