# Supplementary material for: Over the counter use of topical corticosteroid for skin conditions among patients before attending skin specialist clinic in Nepal: A qualitative study
Source: PLOS Glob Public Health. 2025 Jun 24;5(6):e0004812. doi: 10.1371/journal.pgph.0004812 (PMC12186941; doi:10.1371/journal.pgph.0004812)
Supplement: S1_Fig — (PDF) [file pgph.0004812.s004.pdf]

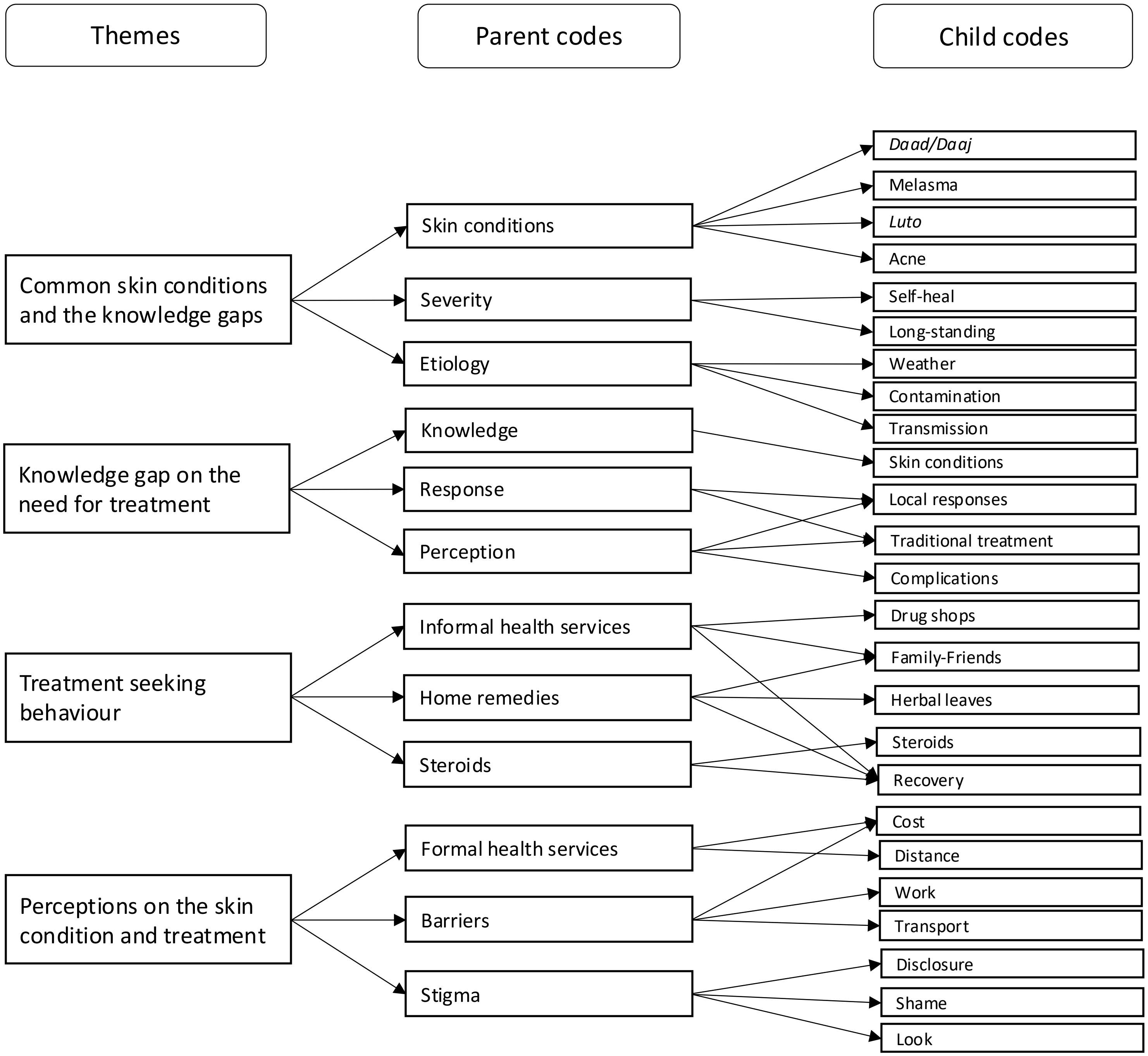
Themes
Parent codes
Child codes
Common skin conditions and the knowledge gaps
Knowledge gap on the need for treatment
Treatment seeking behaviour
Perceptions on the skin condition and treatment
Skin conditions
Severity
Etiology
Knowledge
Response
Perception
Informal health services
Home remedies
Steroids
Formal health services
Barriers
Stigma
Daad/Daaj
Melasma
Luto
Acne
Self-heal
Long-standing
Weather
Contamination
Transmission
Skin conditions
Local responses
Traditional treatment
Complications
Drug shops
Family-Friends
Herbal leaves
Steroids
Recovery
Cost
Distance
Work
Transport
Disclosure
Shame
Look
