# Supplementary material for: Over the counter use of topical corticosteroid for skin conditions among patients before attending skin specialist clinic in Nepal: A qualitative study
Source: PLOS Glob Public Health. 2025 Jun 24;5(6):e0004812. doi: 10.1371/journal.pgph.0004812 (PMC12186941; doi:10.1371/journal.pgph.0004812)
Supplement: Attachment — Submitted filename: PGPH-D-24-02509_reviewer.pdf [file pgph.0004812.s005.pdf]

PLOS Global Public Health

Over the counter use of topical corticosteroid for skin conditions among patients before attending skin specialist clinic in Nepal: a qualitative study.

--Manuscript Draft--

Manuscript Number:	PGPH-D-24-02509
Article Type:	Research Article
Full Title:	Over the counter use of topical corticosteroid for skin conditions among patients before attending skin specialist clinic in Nepal: a qualitative study.
Short Title:	Over the counter use of topical corticosteroids in Nepal.
Corresponding Author:	Yogesh Poudyal, MD<br>Sarnath Skin Centre<br>Bhairahawa, NEPAL
Order of Authors:	Yogesh Poudyal, MD
	Nishika Aryal
	Anuja Rajbhandari
	Mukti Ghimire
	Sanjay Acharya
	Shristi Raut
	Bishal Joshi
	Sunil Pokharel
	Phaik Yeong Cheah
	Bipin Adhikari
Keywords:	Dermatology; Dermatophytosis; Topical Corticosteroids; Nepal
Abstract:	Introduction<br><br>The use of topical corticosteroids (TCs) globally includes over the counter (OTC) sale without proper guidelines, exacerbating treatment-resistant infections and complicating outcome of skin conditions. The main aim of the study was to evaluate the reasons behind OTC use of TCs among patients with skin conditions before attending a skin specialist clinic.<br><br>Methods<br><br>This qualitative study was conducted from September 2021 to March 2022. The study included in-depth interviews (IDIs) and observations, among a total of thirty-one patients with specific skin conditions using TCs, at the Sarnath Skin Centre in Siddhartha Nagar (Bhairahawa), Nepal. The IDIs explored patients' experience with the skin condition, treatment seeking behaviour, OTC medicine experiences including the use of TCs, and consequences. All interviews were conducted in Nepali language, audio-recorded, transcribed and translated in English and were coded and analyzed in NVivo to generate major and minor themes.<br><br>Results<br><br>Among the 31 individuals (12 females and 19 males, aged 18-73 years) diagnosed with Dermatophytosis, Melasma, Eczema, common treatments included use of Sonaderm (Clobetasol, Gentamycin and Miconazole), Derma-KT (Clobetasol, Gentamicin, Ketoconazole and Iodochlorhydroxyquinoline) and steroid-infused products from non-medical sources for prolonged periods of time, exposing patients to transient recovery and perpetuating a vicious cycle of OTC treatment seeking until patients failed to recover. OTC seeking was facilitated by participants' easy access to drug dispensers, suggestions from and practice of family and friends which often led to

	temporary relief for a few weeks/months. In addition, access factors (e.g. distance, cost, time), convenience factors (ease of presenting at the drug store compared to skin clinic) also influenced the choice for OTC. There was a lack of knowledge related to adverse effects of topical steroid, including proper guidance on the use of topical medications and limitations of home remedies.<br><br>Conclusions<br><br>OTC use of topical medications poses insurmountable problems, often developing complicated skin cases presenting late at skin-specialist clinics. A multi-pronged public and community engagement are critical to curb the treatment seeking dynamics among patients developing skin conditions.
Additional Information:	
Question	Response
**Financial Disclosure**<br><br>Enter a financial disclosure statement that describes the sources of funding for the work included in this submission and the role the funder(s) played. This includes grants and any commercial funding of the work or authors.<br><br>This statement will be typeset if the manuscript is accepted for publication.<br><br>*Please review the submission guidelines and the instructions link below for detailed requirements and guidance.*	The study received funding from Society of Dermatologists, Venereologists and Leprologists of Nepal (SODVELON) only adequate for the data collection. SODVELON is an umbrella organization established for the welfare of dermatologists of Nepal. Funding for publication was supported by Mahidol-Oxford Tropical Medicine Research Unit (MORU). The MORU is funded by the Wellcome Trust (220211/Z/20/Z). The study is also part of the foundational work supported by Global Health Bioethics Network (GHBN), UK (228141/Z/23/Z) . BA is a GHBN fellow. For Open Access, the author has applied for a CC BY public copyright license to any Author-Accepted Manuscript version arising from this submission. The funder had no role in the writing or preparation of the manuscript.
**Competing Interests**<br><br>On behalf of all authors, disclose any competing interests that could be perceived to bias this work.<br><br>This statement will be typeset if the manuscript is accepted for publication.<br><br>*Please review the instructions link below and PLOS Global Public Health's competing interests policy to determine what information must be disclosed at submission.*	BA serves as an academic editor of PLOS Global Public Health
**Data Availability**	Data cannot be shared publicly because of the risk that respondents could be

Before publication, Authors are required to make fully available and without restriction all data underlying their findings. Please see our PLOS Data Policy page for detailed information on this policy.<br><br>A **Data Availability Statement**, detailing where the data can be accessed, is required at first submission. Insert your Data Availability Statement in the box below.<br><br>Please see the data reporting section of our submission guidelines for instructions on what you need to include in your Data Availability Statement.<br><br>This statement will be typeset if the manuscript is accepted for publication.<br><br>*PLOS allows rare exemptions to address legal and ethical concerns. If you have legal or ethical restrictions, please use the box below to detail these in full sentences for the Journal team to consider.*	identified. Given the context of the interviews even with identifying information removed, it is relatively easy to identify respondents based on their visit to the clinic and services they received. In addition, participants did not provide broad consent to share their data outside of the study team. However, data are available on reasonable request through email to corresponding author.

Powered by Editorial Manager® and ProduXion Manager® from Aries Systems Corporation

Manuscript Click here to access/download;Manuscript;MANUSCRIPT.docx

Over the counter use of topical corticosteroid for skin conditions among patients before attending skin specialist clinic in Nepal: a qualitative study.

Yogesh Poudyal[1*], Nishika Aryal[2], Anuja Rajbhandari[2], Mukti Ghimire[3], Sanjay Acharya[4], Shristi Raut[5], Bishal Joshi[6], Sunil Pokharel[7], Phaik Yeong Cheah[7,8], Bipin Adhikari[7,8,9]

[1]Sarnath Skin Centre, Shanti Path, Siddhartha Nagar-8, Bhairahawa, Nepal

[2]Department of Public Health, La Trobe University, Melbourne, Australia

[3]Department of Pediatrics, Nepal Police Hospital, Krishnadhara Marg-3, Kathmandu, Nepal.

[4]Academy for Data Science and Global Health, Kathmandu, Nepal

[5]Department of Microbiology, Institute of Medicine, Tribhuvan University, Maharajgunj Campus, Kathmandu, Nepal

[6]Department of Physiology, Universal College of Medical Sciences, Bhairahawa, Nepal

[7]Centre for Tropical Medicine and Global Health, Nuffield Department of Medicine, University of Oxford, Oxford, United Kingdom

[8]Mahidol-Oxford Tropical Medicine Research Unit, Faculty of Tropical Medicine, Mahidol University, Bangkok, Thailand

[9]Central Department of Microbiology, Tribhuvan University, Kirtipur, Nepal

*Correspondence: Dr. Yogesh Poudyal, E-mail: neverland791@yahoo.com

Abstract

Introduction

The use of topical corticosteroids (TCs) globally includes over the counter (OTC) sale without proper guidelines, exacerbating treatment-resistant infections and complicating outcome of skin conditions. The

main aim of the study was to evaluate the reasons behind OTC use of TCs among patients with skin conditions before attending a skin specialist clinic.

Methods

This qualitative study was conducted from September 2021 to March 2022. The study included in-depth interviews (IDIs) and observations, among a total of thirty-one patients with specific skin conditions using TCs, at the Sarnath Skin Centre in Siddhartha Nagar (Bhairahawa), Nepal. The IDIs explored patients' experience with the skin condition, treatment seeking behaviour, OTC medicine experiences including the use of TCs, and consequences. All interviews were conducted in Nepali language, audio-recorded, transcribed and translated in English and were coded and analyzed in NVivo to generate major and minor themes.

Results

Among the 31 individuals (12 females and 19 males, aged 18-73 years) diagnosed with Dermatophytosis, Melasma, Eczema, common treatments included use of Sonaderm (Clobetasol, Gentamycin and Miconazole), Derma-KT (Clobetasol, Gentamicin, Ketoconazole and Iodochlorhydroxyquinoline) and steroid-infused products from non-medical sources for prolonged periods of time, exposing patients to transient recovery and perpetuating a vicious cycle of OTC treatment seeking until patients failed to recover. OTC seeking was facilitated by participants' easy access to drug dispensers, suggestions from and practice of family and friends which often led to temporary relief for a few weeks/months. In addition, access factors (e.g. distance, cost, time), convenience factors (ease of presenting at the drug store compared to skin clinic) also influenced the choice for OTC. There was a lack of knowledge related to adverse effects of topical steroid, including proper guidance on the use of topical medications and limitations of home remedies.

Conclusions

OTC use of topical medications poses insurmountable problems, often developing complicated skin cases presenting late at skin-specialist clinics. A multi-pronged public and community engagement are critical to curb the treatment seeking dynamics among patients developing skin conditions.

Keywords: Dermatology, Dermatophytosis, Topical Corticosteroids, Nepal

Introduction

Topical corticosteroids (TCs) have emerged as the primary choice for the dermatologist in outpatient care more prominently since the 1950s, serving as the fundamental components in dermatologic therapy [1]. TCs are available in the form of cream, ointment, gel, and solution [2]. They are among the most frequently prescribed dermatological medications but their widespread use has become a double-edged sword [3]. Many of these drugs were misused for diverse indications such as pigmentation, acne, pruritus, infections, rashes, and numerous other condition because of the properties they had, such as lightening and anti-inflammatory effects [4].

The abuse of TCs is widespread particularly in Asian and African countries where people prefer lighter skin condition and colorism has profound social impact [5]. This abuse is due to inadequate health infrastructure, limited specialist service, self- medication practices, easy access to over the counter (OTC) medications, incorrect prescriptions, misleading pharmaceutical marketing, and inadequate regulation of irrational drug combinations [6, 7].

Over the counter use of antimicrobials in Nepal remains to be a major problem and previous studies have outlined the reasons behind the OTC purchase of antimicrobials including the surge during the recent COVID-19 pandemic [8-10]. These studies have highlighted the major regulatory gaps in monitoring the OTC use of antimicrobials. The lack of effective oversight in supervising and monitoring medicine shops allows the unrestricted sale of medications [11]. General practitioners and pharmacists frequently recommend these products as they can offer immediate symptomatic relief [12]. In 2013, the top selling topical steroid (TS) combination included beclomethasone, neomycin, and clotrimazole, generating Rs. 152 crores (approx. USD25 million). Despite lacking scientific rationale, these broad-spectrum products dominated the market [4, 13].

In Nepal, many topical preparations are sold in pharmacies as OTC medications (the price could range from 0.5 USD to 4 USD), i.e. these can be given to patients without doctor's prescription and there are no definite guidelines for OTC use and prescription medications [14]. Dermatophytosis is a common disorder of skin also commonly known as ringworm and is a fungal infection of the skin, hair, or nails [15]. Among skin conditions, dermatophytosis has long been one of the most common skin infections, the significant shift in clinical profile, with rise in chronic, recurrent , or treatment resistant cases is concerning [16]. This resistance poses a major challenge to achieving a complete cure. Easy availability and widespread uses of irrational combination cream containing potent steroid, anti-fungal and anti-

bacterial are deemed to be one of the most significant reasons for alarming outbreak of Tinea and recalcitrant type of dermatophytosis [17, 18]. Moreover in Nepal, dermatology clinics frequently deal with steroid induced and steroid aggravated dermatosis [19]. Managing these complications is often more difficult and distressing for patients than treating the original condition for which steroids were inappropriately used [20]. In more than 93% of the skin cases, TC is used unnecessarily for an extended duration beyond requirement, of inappropriate potency or administered without a thorough diagnosis of underlying condition [11].

There are plenty of studies which highlight the burden of topical steroid misuse [4, 12, 20-22]. However, there are limited studies exploring rationales behind the steroid use among dermatology patient, deriving from the patients' living experience with the skin condition and treatment seeking behaviors. The main objective of this study was to explore the patient's experience of dermatological conditions, the treatment seeking behavior including decision to use OTC steroid topical preparation and the consequences.

Methods

Design and overview

This was a phenomenological study that explored a particular phenomenon under the research question to explore the experience and response to it. The particular phenomenon in this study refers to the skin condition patients have developed and sought treatment at the dermatological clinic served by the first author (YP). To explore the phenomenon under the study, data were collected using in-depth interviews (IDIs) and observations of patients with skin conditions. The specific type of patients (respondents) for this study comprised patients with any skin diseases that made them use topical steroids. Grounded in the philosophical tradition of phenomenology, this study aimed to understand participants' lived experiences and their encounters with steroid use and skin conditions. The study followed a COREQ (Consolidated criteria for Reporting Qualitative research) guideline (**S1 File**).

Study settings

This study was conducted in Sarnath Skin Centre, a skin specialist clinic located in the urban centre of Bhairahawa. Sarnath clinic was established in 2017 AD and serves around 40 skin specific patients a day. Bhairahawa, officially recognized as 'Siddharthanagar' is a municipality and the administrative center of

Rupandehi district in Lumbini province of Nepal. The city is a border town with India on the southern plains of Western Nepal with a population of around 75,000 people [23]. The city has a tertiary hospital hosted by Universal College of Medical Sciences, private clinics, drug shops and specialist clinic.

Bhairahawa as an administrative and urban center of the district receives patients from the settlements around the urban center including patients from northern mountains who visit for specialist clinic care. Most of the patients attending the specialist clinic came from outskirts of the urban Bhairahawa and were mostly working in the farmlands, particularly rice fields.

Selection of respondents

All respondents were selected purposively in this study, and the total number of participants was determined based on data saturation whereby no new data emerged from the further interviewing of the patients [24]. Patients with specific skin conditions with prior history of use of topical corticosteroid were selected to align with the research question of this study. Participants were first verbally asked if they were interested to participate in a study during their clinical examination. Once they verbally agreed, a written informed consent was obtained and were interviewed in the separate room within the clinic premise. No repeat interviews were conducted, and each interview lasted around 45minutes to 1 hour. The study was conducted between September 2021 and March 2022. A total of 31 patients were interviewed including ten non-participant observations based at the drug shop. Non-participant observation notes were written by the first author on skin patients' interactions with the dispenser and their decision-making dynamics before they attended the dermatologist.

The interviews were conducted by YP with participants aged 18 years and above. YP is a dermatologist (MBBS, MD) with more than 10 years of dermatological practice in the region. Individuals who were not physically or mentally fit, or unwilling to provide written informed consent were respected and excluded from the study. Confidentiality of the data was maintained throughout the research process.

Data collection guide

Data were collected using IDI guide that included a range of topics incorporating patients' experiences regarding their skin conditions, normative health-seeking behavior, knowledge of OTC and TC, and policies related to TC (**S2 File**). The thematic guide for the interviews was prepared and reviewed by the authors (YP and BA). All interviews were conducted in Nepali and recorded using an audio recorder, with additional non-verbal cues and relevant information noted during the interviews and were documented

as field notes. Subsequently, all recorded interviews were translated and transcribed into English for analysis.

Data Analysis

Participants' personal information was anonymized to protect their identity. All transcribed data were stored in Microsoft Word and then analyzed by two coders (NA and AR) using NVivo software. A mix of deductive and inductive approaches to coding of data were utilized which informed the thematic synthesis and was critically reviewed by BA, YP and BJ. The analyzed data were thoroughly cross-checked for any mis-association and misinterpretations. By focusing on the subjective experiences of the participants, the research sought to uncover deeper meanings and insights into their personal events.

Ethical approval

This study received ethical approval from Nepal Health Research Council (NHRC Ref # 388, August 27, 2021). All respondents provided written informed consent to participate in the study.

Results

The socio-demographic details of the participants are presented in table 1. Most of the participants lived within a radius of 20 km from clinic site. Four broad themes were identified that included a) skin conditions and the participants knowledge, b) knowledge gap; and c) treatment seeking behavior; d) patient perception (**Figure 1**). These four broad themes were further consolidated into two major themes that included 1. Skin conditions and the knowledge gaps; and 2. Treatment seeking behavior.

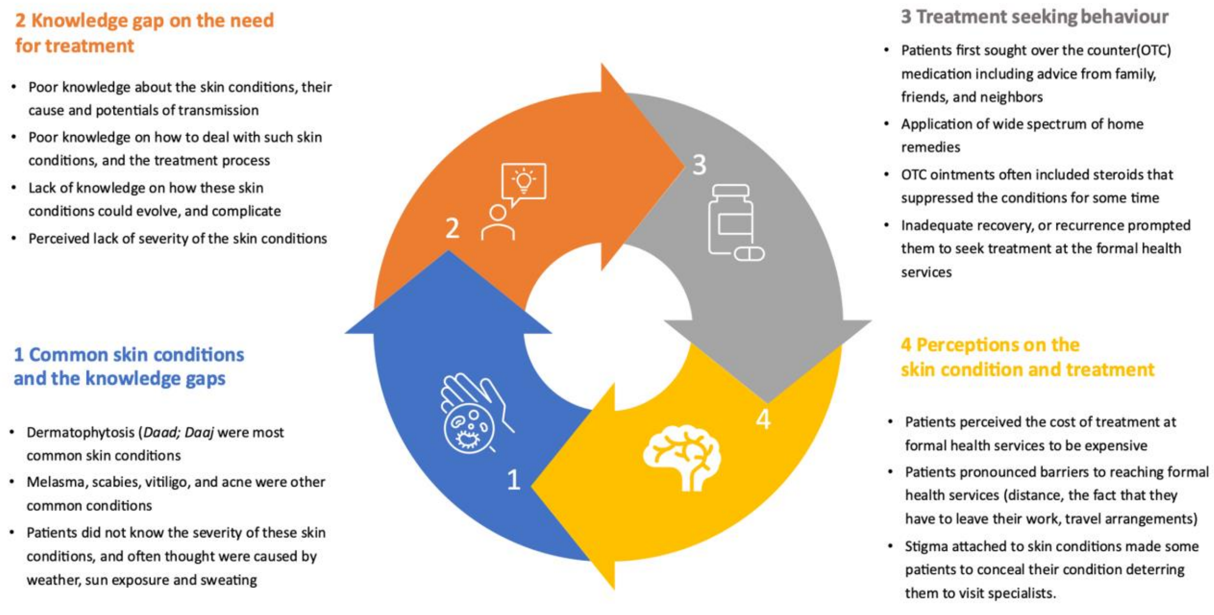

Figure 1: Major themes and some of the prominent findings.

**Table 1:** Socio-demographics of the participants in the study					
**S. N.**	**Age**	**Sex**	**Educational level**	**Profession**	**Diagnosis**
1	40-50	F	Grade 10	Housewife	Dermatophytosis
2	20-30	M	Grade 10	Job	Dermatophytosis
3	20-30	F	Grade 10	Housewife	Dermatophytosis
4	30-40	M	Grade 10	Business	Dermatophytosis
5	18-20	M	+2 Level	Student	Dermatophytosis
6	20-30	F	Grade 9	Farmer	Dermatophytosis
7	20-30	F	Grade 3	Housewife	Dermatophytosis
8	18-20	M	Grade 10	Student	Dermatophytosis
9	30-40	F	+2 Level	Job	Dermatophytosis
10	40-50	M	Grade 10	Job	Dermatophytosis
11	20-30	F	Bachelor level	Student	Dermatophytosis
12	30-40	M	Grade 6	Job	Dermatophytosis
13	20-30	M	Bachelor level	Job	Dermatophytosis
14	20-30	M	Bachelor level	Job	Dermatophytosis
15	20-30	F	Grade 8	Housewife	Melasma
16	18-20	M	Grade 10	Student	Dermatophytosis
17	18-20	M	+2 Level	Student	Dermatophytosis

18	70-80	M	Grade 10	Business	Dermatophytosis
19	18-20	M	+2 Level	Student	Dermatophytosis
20	50-60	M	Diploma	Job	Dermatophytosis
21	20-30	M	Grade 10	Farmer	Dermatophytosis
22	20-30	M	+2 Level	Businessman	Dermatophytosis
23	18-20	M	+2 Level	Student	Dermatophytosis
24	20-30	M	Master level	Student	Eczema
25	20-30	M	Grade 10	Farmer	Eczema
26	30-40	M	Grade 10	Job	Dermatophytosis
27	40-50	F	Grade 10	Job	Dermatophytosis
28	20-30	F	Grade 10	Housewife	Melasma
29	30-40	F	+2 Level	Housewife	Melasma
30	30-40	M	Grade 10	Businessman	Dermatophytosis
31	40-50	M	Bachelor level	Job	Dermatophytosis

F: Female, M: Male, most common variant of dermatophytosis was T. incognito

Job: working in small business firm or private institute with fixed monthly salary.

Dermatophytosis: Superficial fungal infection of skin, hair and nail caused by dermatophyte.

Eczema: it is skin condition characterized by variable intensity of itching and soreness, and in variable degree signs including dryness, redness, excoriation, exudation and fissuring

Melasma: it is manifested as hyperpigmented patch on the face that become more pronounced after sun exposure.

Skin conditions and the knowledge gaps

Daaz, Daad [dermatophytosis] was the most common skin condition reported by participants later confirmed by the dermatologist. Other frequently encountered skin issues in their locality were melasma, scabies, vitiligo, and acne. These conditions represented a significant portion of the dermatological problems faced by the community.

> *I don't exactly know the name, but in our language, we call it "Daad". Only Daad is prevalent in our family members and locality. – Pt-22, 20-30 years, male.*

Most of the participants associated their skin condition with factors such as hot weather, sun exposure, and sweating. They believed that these environmental and personal habits were primary cause of their skin conditions. Their overall basic knowledge about skin diseases was very poor. Even for diseases like dermatophytosis, which affected almost all the members of household, they even didn't realize its infective nature.

> *Skin conditions are due to the use of wet clothes, wet underwear and due to sweat and if we don't bathe regularly. – Pt-16, 18-20years, male*

People did not have sufficient knowledge regarding their skin diseases and its treatment process. Whenever they were affected by any condition, they were unaware about where and with whom to receive healthcare services. It was common thinking among the participants that their disease was not complicated, not knowing about its potential risks and haphazardly consuming drugs rather than actual treatment from a certified specialist. But after a prolonged prevalence and recurrence, they realized that this disease was not as common as they thought it to be.

> *ya at first, I used to think it simple…….in initial phase it used to get cured after applying ringguard …… so I took it simply……but now when it kept on repeating for so many years I thought of getting consultation. – Pt-31, 40-50 years, male*

Due to this misinformation and knowledge gap, people practiced some unusual home remedies like using goat's urine, Harpic (hydrochloric acid), detergent powder, and even considered the path of invocation which was extremely concerning.

Some people even suggested using goat urine, but that didn't work either. Then there were recommendations to use bitter gourd and camphor, so I tried mixing those as well, but nothing worked. Eventually, I became tired of applying anything at all. – Pt- 30, 30-40 years, Male.

Patients were not properly instructed by the medical shop owner about the necessity of a prescription for medications. Another common phenomenon was that the medical shop owner/ pharmacist normally provided medicines to the participants after they demanded it without inspecting and physically seeing their skin disease

….He used to ask what happened…..and I used to say "daaz"….than I used to ask for ringguard,.. and he used to give it immediately. – Pt-31, 40-50 years, male

Treatment seeking behavior

Observations at the skin clinic's pharmacy revealed that patients often wavered between consulting the specialist or trying over-the-counter medications first. Positive counseling from the dispenser about the benefits of seeing a dermatologist influenced many of their decisions. However, some patients opted to see the specialist without needing further discussion. Some of these patients who had pre-decided to see the skin specialist were either having long standing skin conditions or were not cured by their repeated use of OTC use of medications.

Initially, when individuals noticed symptoms of their skin condition, they either went to a medical shop to get medication without a prescription or relied on advice from family, friends, neighbors, and relatives. They even applied home remedies or medications in the form of ointment, cream, gel, which temporarily eased their symptoms, allowing them to return to their usual activities. However, after a few weeks or months, the condition recurred making them obtain the same treatment from the pharmacy. This cycle was repeated until they either became exhausted or their condition worsened significantly. This pattern illustrated their coping mechanism of relying on pharmacists for symptom management whenever their skin issues reappeared. Moreover, it was common for people in local communities to heavily rely on medical shop owners for medical advice and treatment, despite the lack of formal

medical training. This habit underscored the accessibility and convenience of medical shops, which often served as the first point of contact in healthcare for many individuals.

> *I got advice from the villagers who had suffered from similar disease earlier regarding how their disease got cured. I followed their advice. Then I thought, if I didn't cure then I visited a skin doctor. – Pt-19, 18-20 years, male*

> *While using the medicine my face became very dry, it removed the black spots, but it looked as if the moisture from my face had disappeared. So, I stopped using that medicine. After that, I went again for an incantation (to invoke a magical effect or influence) through which I got some relief. I used aloe vera for 10-20 days, but nothing happened. Later on, one person gave me the address of this clinic. – Pt-29, 30-40 years, male*

Commonly used medicines for skin condition among participant included Sonaderm (clobetasol propionate, gentamycin sulphate, miconazole nitrate), Derma-KT (ketoconazole, neomycin sulphate, Iodochlorohydroxyquinoline, tolnaftate, clobetasol propionate), Derma-5 (clobetasol propionate, gentamycin, iodochlohydroxyquinoline, ketoconazole) , Mofur (mometasone), quadriderm (clotrimazole, neomycin, beclomethasone) , Melalite (hydroquinone, mometasone, tretinoin), Ring Guard(miconazole nitrate, neomycin sulphate, chlorocerol), SkinLite(Hydroquinone, mometasone, tretinoin). SkinLite was also found in non-medical settings like fancy shop or cosmetic shops. It seemed they were used as cosmetics without understanding its potential side effects. Almost all of them were not aware of its composition and its potential side effects.

> *Everything is available in the fancy shops. In Mahilwar, every shop has Chinese cream, and night cream like Melalite, and SkinLlite. I have a brother who owns a medical store. He gave me SkinLite and told me, "Sister, use this cream and you'll glow like a foreigner" and I used that cream and my skin became fair. But my skin was damaged with time. I used that for about 2-3 months. – Pt-15, 20-30 years, female*

Patients used to delay in seeking specialist care for their skin conditions due to various factors, such as the distance, time required to reach healthcare facilities, financial constraints, and the temporary relief provided by medications obtained from pharmacies. The long and significant travel distance to the clinic posed substantial difficulties for both first-time patients and those requiring regular visits. Most people relied on local transportation and bikes to reach the clinic, adding to the challenge of accessing necessary medical care.

Today I also came all the way from Rajahar (approx. 80 km from the interview site) because I have some work over here and relatives here told me to get a checkup from you. – Pt-4, 30-40 years, male

I used medicine from India for six months, I used to travel to India every 15 days. – Pt- 29, 30-40 years, female

Additionally, some patients were unaware of where to find dermatologists, while others felt embarrassed about their condition. However, some individuals prioritized finding an effective cure, even if the expenses were to be high, as they were tired by the recurrent nature of their skin problems. In the absence of professional medical guidance, patients often resorted to alternative remedies like turmeric, garlic, neem, aloe vera, camphor, alum, herbal juices, and bean leaves, which provided some temporary relief but did not address their underlying cause of the skin condition. Some of them also watched various advertisements on TV and the internet and tried the remedies/medication suggested for their problem.

When I first noticed this disease, I thought it was just a simple irritation of skin folds and I applied a powder for 2-3 days. But nothing changed and that area became red. There were small red dots, and I thought about what to do with it. Then, one friend told me it is a simple daaz. I remember watching ring guard advertisements on TV. I searched for it, bought it and applied. – Pt-31, 40-50 years, male

The excessive and prolonged use of TCs raised concerns, including diminished efficacy due to tachyphylaxis, skin atrophy, and steroid-induced dermatoses. Patients, often in an attempt to manage chronic dermatological conditions, resorted to applying TCs creams and ointments far beyond recommended dosages and durations.

I kept on applying those tubes 7-8 times after an interval of some months that is around 25-30 tubes for the whole 2 years. – Pt-26, 30-40, male

Despite receiving immediate healthcare from specialist doctors on several occasions with multiple consultations, the patient continued to experience recurring issues with their condition. This indicated

more prevalence of recalcitrant type of dermatophytosis which is not even cured even with standard treatment regimen, making treatment long and costly. This has made patients lose faith in specialist consultation and is again fueling steroid abuse.

In the past two years, I went to the medical college and underwent some tests, but my condition still did not improve. I visited the doctor 2-3 times for regular follow-ups, and my condition completely disappeared, making me feel that the disease was gone. However, when it reappeared, I started to think that either the medication was incorrect or the doctor prescribed it without proper evaluation. I believed that even consulting a doctor wouldn't help, so I continued taking the same medication repeatedly. – Pt-3, 20-30 years, female

Participants perceived that visiting a medical shop was more convenient, cheaper, and less time-consuming due to the lack of access to nearby doctors or information on where to find certified medical professionals. People who worked in medical shops and provided medications were informally referred to as "doctors," despite not necessarily holding medical degrees or qualifications. Many were unaware of the benefits of being treated by a certified doctor and often could not allocate time for a proper medical visit. Consequently, they adapted to using medicines from nearby medical shops, experiencing only temporary relief rather than a cure.

Participants also tended to hide their skin conditions out of fear of social rejection and shame. However, after suffering for extended periods, some were persuaded by close ones to visit a specialist, particularly those who themselves had been successfully treated by a dermatologist. For others, unbearable pain eventually compelled them to seek professional help. Upon visiting a specialist, they realized they had been using unnecessary medications and ointments and discovered that their conditions could be permanently cured with appropriate treatment.

We refer to the individuals who work in medical shops and dispense medications as "doctors”. – Pt-23, 18-20 years, male.

They might feel ashamed because daaz often appears in areas like the groin. – Pt- 24, 20-30 years, male

Discussion

The study explored the OTC use of topical corticosteroid among patients attending outpatient clinics in Bhairahawa, with dermatophytosis being the most common issue alongside other conditions such as melasma, and Acne. Participants often associated environmental factors and inadequate hygiene practices as the causes of these conditions, leading them to frequently resort to self-medications. The delay in seeking specialist care was influenced by various factors including distance, cost, and lack of awareness about proper treatment. Misuse of medications containing TC compounds was also observed, contributing to the side effects and prolonged suffering. Knowledge gap regarding skin diseases and their treatment processes were evident. Despite efforts to address these issues, challenges persisted due to a lack of steroid awareness, improper medication guidance, and a preference for convenience over specialist care.

Most case of steroid misuse noted in this study was dermatophytosis. Tinea incognito and acne were most frequent complications noted in patients misusing steroid in India [6]. A study by Molly et al [12] showed 58% of the participant perceived their skin condition to be allergic to food when in fact 70.1% were Tinea. One study in Bhairahawa showed that those who consulted dermatologist were less likely to misuse steroids [25]. Our study also showed participants had poor knowledge of skin disease. This might have paved the way for steroid misuse.

Management of skin diseases among our participants often seem to start with home remedies, followed by seeking advice from family circle and friends. If these early treatments failed, they seem to seek advice and medicine from nearby pharmacy. After long duration of trial and error with the OTC steroid often without recovery, or worst when they developed complications, they finally seek treatment at the specialist clinics. Study by Saraswat et al [11] showed that more than half of patients received recommendation to use TC from non-physician source such as peers or relatives, and drug dispensers. Similarly, drug dispensers and local healer contributed to 78% contribution to the OTC use of steroid. Relying on non-physician source for the treatment was one of the important causes of OTC use of steroid.

Patients frequently avoided consulting dermatologists due to time constraints or because they see others using the same medications for similar skin issues [26]. Research in Australia revealed that individuals experiencing skin conditions reported increased self-consciousness, heightened embarrassment, and feelings of social exclusion [27]. A study in Nepal found that the lack of awareness

and the shortage of dermatologists, especially in rural areas, led to increased OTC medication use [20]. Similarly, our study observed that individuals often went directly to drug dispensers because of the unawareness about the presence of dermatologist in their reach or sometimes simply because of lack of knowledge on who to consult.

In our study, most people when asked on why they did not visit specialist care, they seem to be unaware of the concept and benefits of health insurance. Similarly, data from the Government of Nepal in 2021 showed that approximately 29% of families were unaware of the health insurance policy, and 34.1% were aware but did not enroll [28]. Additionally, limited healthcare coverage and high rates of infectious diseases in Nepal [29, 30], led many to rely on OTC medication [31, 32]. Pharmacies/drug shops were popular due to their accessibility, low cost, and convenience, despite offering lower quality care. These medical shops were preferred because they involved minimal direct, indirect, and opportunity costs, making them an attractive option for many individuals [8].

The abundance of online information posed a risk, as patients often couldn't distinguish between credible and unreliable sources. For example, a teenager with acne might find home remedies such as applying apple cider vinegar or toothpaste, leading to burns [33]. This issue echoes with our study, where participants reported using unconventional and potentially harmful remedies such as goat's urine and *Harpic* (a toilet cleanser solution) for their skin conditions. Skin diseases were often underestimated with attitudes that were expressed as *"It's just a rash"* or *"it's not life-threatening"*. However, only recently has there been recognition of the substantial impact that chronic, disfiguring, or disabling skin diseases had on the quality of life of patients and their families [34]. Additionally, there was a common practice of reusing old prescriptions for recurrent or new lesions [35], and echoes with our participants.

The research highlights the significant problem of steroid misuse evading treatment seeking at the skin specialists and has been echoed across many countries such as Iraq, Nepal and India where a substantial percentage of dermatology outpatients were improperly using TCs [17, 36, 37]. In India, misuse of TS on the face has become a major concern, leading to conditions like steroid addiction or red face syndrome. This results in severe rebound erythema, burning sensations, and scaling when TS use is discontinued after long-term use [11].

In our study, participants reported experiencing similar side effects after using TC, such as a burning sensation, redness, and thinning of the skin. Concerning side effects, dosage, and proper usage instructions, 97.9% of patients in India were unaware of the potential adverse effects of TS and had not read the manufacturer's leaflet [35]. In Saudi Arabia, research showed low awareness of corticosteroid

side effects among both users and non-users, with users slightly more informed [38]. Similarly, participants in our study were unaware of steroids and had never read the medication composition even when they were found to use it for a long time.

Implications

The study highlights the high OTC use of topical corticosteroid and thus warrants urgent sets of efforts to curb the treatment seeking behaviour. First, community and public engagement efforts are critical to enhance the awareness of skin conditions and the rationale for consulting physicians which could safeguard the ethics and interests of various stakeholders [39, 40]. The potential consequences of delayed treatment seeking behaviour [41-43] as outlined in this study further highlights the addition of both direct and indirect costs, stress, time and resources on both supplier and provider sides. Exploring these dimensions through health economics study could add to the findings of this study. Second, the widespread use of topical corticosteroid, including advertisements over media on beauty products require immediate scrutiny so that these products are regulated. The OTC use of these products is highly likely to trigger the concomitant use of antimicrobials and thus antimicrobial resistance interventions require a concerted effort. Third, engagement with dispensers, pharmacies (drug shops) and stakeholders are critical to promote their understanding of the OTC use of steroids, beauty products and antimicrobials and their consequences.

Strengths and limitations of study

The study included specific respondents with skin conditions with history of using TCs for variable period, thus may not address the treatment seeking behaviour for other general skin conditions. These respondents had rich lived experiences and thus the insights from these respondents highlight the scenario of skin conditions among patients including factors influencing topical steroid misuse. Nonetheless, by virtue of selecting these specific patients we may have incurred selection bias. Furthermore, as this is a clinic-based study only those cases with symptoms secondary to steroid abuse were recruited, potentially overlooking asymptomatic instances of steroid misuse.

Conclusions

Lack of knowledge about skin diseases, perceived lack of severity and reliance on friends, family and home remedies at the initial stage seem to have delayed the treatment. Lack of recovery and

development of complications often made these patients to seek OTC medications; and when these medications as well did not cure their skin conditions, patients sought treatment at the formal health services including dermatologists. The barriers in reaching formal health services, specialist clinic and most importantly patients' disposition to underestimate skin conditions seem to have affected the outcome of these skin conditions. An early treatment seeking at formal health services (specialist care) is critical to ensure that these patients save resources and receive appropriate treatment before the development of consequences. A multifaceted approach embedded in public and community engagement is essential to address the treatment-seeking behaviors of patients with skin conditions.

Declarations

Abbreviations

TCs- Topical Corticosteroid, OTC- over the counter, TS- Topical Steroid, IDI- in- depth interview, COREQ: Consolidated Criteria for Reporting Qualitative Research.

Acknowledgements

We are grateful to the respondents who generously provided additional time when visiting clinics and insights for this study.

Funding

The study received funding from Society of Dermatologists, Venereologists and Leprologists of Nepal (SODVELON) only adequate for the data collection. SODVELON is an umbrella organization established for the welfare of dermatologists of Nepal. Funding for publication was supported by Mahidol-Oxford Tropical Medicine Research Unit (MORU). The MORU is funded by the Wellcome Trust (220211/Z/20/Z). The study is also part of the foundational work supported by Global Health Bioethics Network (GHBN), UK (228141/Z/23/Z) . BA is a GHBN fellow. For Open Access, the author has applied for a CC BY public copyright license to any Author-Accepted Manuscript version arising from this submission. The funder had no role in the writing or preparation of the manuscript.

Authors' contributions

YP and BA conceptualized the study; YP collected data; YP, NA, AR, and BA analyzed the study. YP and BA wrote the first draft contributed by NA and AR followed by all other authors. All authors read and approved the final version of the manuscript.

Availability of data and materials

Data cannot be shared publicly because of the risk that respondents could be identified. Given the context of the interviews even with identifying information removed, it is relatively easy to identify respondents based on their visit to the clinic and services they received. In addition, participants did not provide broad consent to share their data outside of the study team. However, data are available on reasonable request through email to corresponding author.

Ethics approval and consent to participate

This study received ethical approval from Nepal Health Research Council (NHRC Ref # 388, August 27, 2021)

Consent for publication

Not applicable.

Competing interests

The authors declare that they have no competing interests. BA serves as an academic editor of PLOS Global Public Health.

Figure legends

Fig 1: Major themes and some of the prominent findings.

Supporting Information

Supporting Information File S1: COREQ: COnsolidated criteria for REporting Qualitative research

Supporting Information File S2: Interview guide adapted for the study

Supporting Information File S3: Inclusivity in Global Health

References

1. Das A, Panda S: **Use of topical corticosteroids in dermatology: an evidence-based approach.** *Indian journal of dermatology* 2017, **62:**237-250.
2. Jain S, Mohapatra L, Mohanty P, Jena S, Behera B: **Study of clinical profile of patients presenting with topical steroid-induced facial dermatosis to a tertiary care hospital.** *Indian Dermatology Online Journal* 2020, **11:**208-211.
3. Kumar S, Goyal A, Gupta YK: **Abuse of topical corticosteroids in India: Concerns and the way forward.** *Journal of Pharmacology and Pharmacotherapeutics* 2016, **7:**1-5.
4. Nagesh T, Akhilesh A: **Topical steroid awareness and abuse: A prospective study among dermatology outpatients.** *Indian journal of dermatology* 2016, **61:**618-621.
5. Varshney I, Amin SS, Adil M, Mohtashim M, Bansal R, Khan HQ: **Topical corticosteroid abuse–risk factors and consequences.** *Indian Journal of Clinical Dermatology| Volume* 2019, **2**.
6. Meena S, Gupta LK, Khare AK, Balai M, Mittal A, Mehta S, Bhatri G: **Topical corticosteroids abuse: A clinical study of cutaneous adverse effects.** *Indian journal of dermatology* 2017, **62:**675.
7. Srivastava A: **A clinicoepidemiological study of topical corticosteroid misuse at a tertiary care center.** *Journal of Dermatological Treatment* 2019.
8. Adhikari B, Pokharel S, Raut S, Adhikari J, Thapa S, Paudel K, G CN, Neupane S, Neupane SR, Yadav R, et al: **Why do people purchase antibiotics over-the-counter? A qualitative study with patients, clinicians and dispensers in central, eastern and western Nepal.** *BMJ Glob Health* 2021, **6**.
9. Dhungel B, Thapa Shrestha U, Adhikari S, Adhikari N, Bhattarai A, Pokharel S, Karkey A, Limmathurotsakul D, Ghimire P, Rijal KR, et al: **Use of antimicrobials during the COVID-19 pandemic: A qualitative study among stakeholders in Nepal.** *PLOS Glob Public Health* 2023, **3:**e0002166.
10. Rijal KR, Banjara MR, Dhungel B, Kafle S, Gautam K, Ghimire B, Ghimire P, Dhungel S, Adhikari N, Shrestha UT, et al: **Use of antimicrobials and antimicrobial resistance in Nepal: a nationwide survey.** *Sci Rep* 2021, **11:**11554.
11. Saraswat A, Lahiri K, Chatterjee M, Barua S, Coondoo A, Mittal A, Panda S, Rajagopalan M, Sharma R, Abraham A: **Topical corticosteroid abuse on the face: A prospective, multicenter study of dermatology outpatients.** *Indian journal of dermatology, venereology and leprology* 2011, **77:**160.
12. Thomas M, Wong CC, Anderson P, Grills N: **Magnitude, characteristics and consequences of topical steroid misuse in rural North India: an observational study among dermatology outpatients.** *BMJ open* 2020, **10:**e032829.
13. Verma SB: **Sales, status, prescriptions and regulatory problems with topical steroids in India.** *Indian Journal of Dermatology, Venereology and Leprology* 2014, **80:**201.

14. Jha AK, Karki S, Jha SM: **Topical corticosteroid abuse in Nepal: scenario.** In *A Treatise on Topical Corticosteroids in Dermatology: Use, Misuse and Abuse.* Springer; 2017: 189-196

15. Achterman RR, White TC: **Dermatophytes.** *Current Biology* 2013, **23:**R551-R552.

16. Rajagopalan M, Inamadar A, Mittal A, Miskeen AK, Srinivas C, Sardana K, Godse K, Patel K, Rengasamy M, Rudramurthy S: **Expert consensus on the management of dermatophytosis in India (ECTODERM India).** *BMC dermatology* 2018, **18:**1-11.

17. Verma S, Zouboulis C: **Indian irrational skin creams and steroid-modified dermatophytosis–an unholy nexus and alarming situation.** *Journal of the European Academy of Dermatology and Venereology* 2018, **32:**e426-e427.

18. Dogra S, Uprety S: **The menace of chronic and recurrent dermatophytosis in India: Is the problem deeper than we perceive?** , vol. 7. pp. 73-76: Medknow; 2016:73-76.

19. Parajuli S, Paudel U, Poudyal AK, Pokhrel DB: **A clinical study of steroid induced dermatoses.** *Nepal Journal of Dermatology, Venereology & Leprology* 2018, **16:**12-16.

20. Shrestha S, Joshi S, Bhandari S: **Prevalence of misuse of topical corticosteroid among dermatology outpatients.** *JNMA: Journal of the Nepal Medical Association* 2020, **58:**834.

21. Jaccob AA, Yaqoub AA, Rahmani MA: **Impact of abuse of topical corticosteroids and counterfeit cosmetic products for the face: prospective demographic study in basrah city, Iraq.** *Current Drug Safety* 2020, **15:**25-31.

22. Chaudhary RG, Rathod SP, Jagati A, Baxi K, Ambasana A, Patel D: **Prescription and usage pattern of topical corticosteroids among out-patient attendees with dermatophyte infections and its analysis: A cross-sectional, survey-based study.** *Indian Dermatology Online Journal* 2019, **10:**279-283.

23. Population C: **Siddharthanagar. Available online at https://bit.ly/4eTikVi (accessed on 6th October 2024).** 2024.

24. Saunders B, Sim J, Kingstone T, Baker S, Waterfield J, Bartlam B, Burroughs H, Jinks C: **Saturation in qualitative research: exploring its conceptualization and operationalization.** *Qual Quant* 2018, **52:**1893-1907.

25. Poudyal Y, Joshi SD: **Medication practice of patients with dermatophytosis.** *JNMA J Nepal Med Assoc* 2016, **55:**7-10.

26. Paudel S, Parajuli N, Dahal SC, Paudel S: **Improper use of topical corticosteroids in tinea infections in a tertiary care hospital.** 2021.

27. Magin P, Adams J, Heading G, Pond D, Smith W: **Experiences of appearance-related teasing and bullying in skin diseases and their psychological sequelae: results of a qualitative study.** *Scandinavian journal of caring sciences* 2008, **22:**430-436.

28. Thapa R, Lamsal S, Badhu A, Shrestha S: **Awareness regarding health insurance policy scheme of government of Nepal among local residents of dharan sub-metropolitan city.** *Journal of BP Koirala Institute of Health Sciences* 2021, **4:**32-36.

29. Mishra SR, Khanal P, Karki DK, Kallestrup P, Enemark U: **National health insurance policy in Nepal: challenges for implementation.** *Global health action* 2015, **8:**28763.

30. Rai SK: **Changing trend of infectious diseases in Nepal.** In *Infectious Diseases and Nanomedicine III: Second International Conference (ICIDN-2015), Dec 15-18, 2015, Kathmandu, Nepal*Springer; 2018: 19-38.

31. Pokharel S, Adhikari B: **Antimicrobial resistance and over the counter use of drugs in Nepal.** *J Glob Health* 2020, **10:**010360.

32. Pokharel S, Raut S, Adhikari B: **Tackling antimicrobial resistance in low-income and middle-income countries.** *BMJ Glob Health* 2019, **4:**e002104.

33. Mehta-Ambalal SR, Nisarta M: **Dermatology 2.0-how the internet is changing us, our patients and our practice.** *Indian Dermatology Online Journal* 2021, **12:**593-596.

34. Koblenzer CS: **The emotional impact of chronic and disabling skin disease: a psychoanalytic perspective.** *Dermatologic clinics* 2005, **23:**619-627.

35. Sheth NK, Nair PA: **Topical steroids: Awareness and misuse among patients, pharmacists and general medical practitioner.** *Indian Journal of Dermatology, Venereology and Leprology* 2021, **87:**54-59.

36. Hameed AF: **Steroid dermatitis resembling rosacea: a clinical evaluation of 75 patients.** *International Scholarly Research Notices* 2013, **2013:**491376.

37. Ahmed G, Mishra DK: **A hospital-based observational study on the frequency of different skin diseases and patterns of topical steroid misuse.** *Indian Journal of Drugs in Dermatology* 2018, **4:**67-72.

38. Al-Jabr KH, Alhumaidan LS, Alghamdi AA, Almutairi MSL, Alsubaihi AA, Alrasheedi SM, Alkhdairi A, Alzweihary AM, Alrasheedi MS, Alrasheedi KAM: **Awareness of Side Effects of Corticosteroids among Users and Nonusers in Saudi Arabia.** *Journal of Pharmacy and Bioallied Sciences* 2024, **16:**S1612-S1618.

39. Pokharel S, Adhikari B, Johnson T, Cheah PY: **Interventions to address antimicrobial resistance: an ethical analysis of key tensions and how they apply in low- income and middle-income countries.** *BMJ Glob Health* 2024, **9**.

40. Adhikari B, Pell C, Cheah PY: **Community engagement and ethical global health research.** *Glob Bioeth* 2020, **31:**1-12.

41. Mshamu S, Meta J, Sanga C, Day N, Mukaka M, Adhikari B, Deen J, Knudsen J, Pell C, von Seidlein L: **Care seeking for childhood illnesses in rural Mtwara, south-east Tanzania: a mixed methods study.** *Trans R Soc Trop Med Hyg* 2024.

42. Adhikari B, Phommasone K, Pongvongsa T, Koummarasy P, Soundala X, Henriques G, Sirithiranont P, Parker DM, von Seidlein L, White NJ, et al: **Treatment-seeking behaviour for febrile illnesses and its implications for malaria control and elimination in Savannakhet Province, Lao PDR (Laos): a mixed method study.** *BMC Health Serv Res* 2019, **19:**252.

43. Marahatta SB, Yadav RK, Giri D, Lama S, Rijal KR, Mishra SR, Shrestha A, Bhattrai PR, Mahato RK, Adhikari B: **Barriers in the access, diagnosis and treatment completion for tuberculosis patients in central and western Nepal: A qualitative study among patients, community members and health care workers.** *PLoS One* 2020, **15:**e0227293.

Figure 1

Click here to access/download;Figure;FIGURE 1.pptx

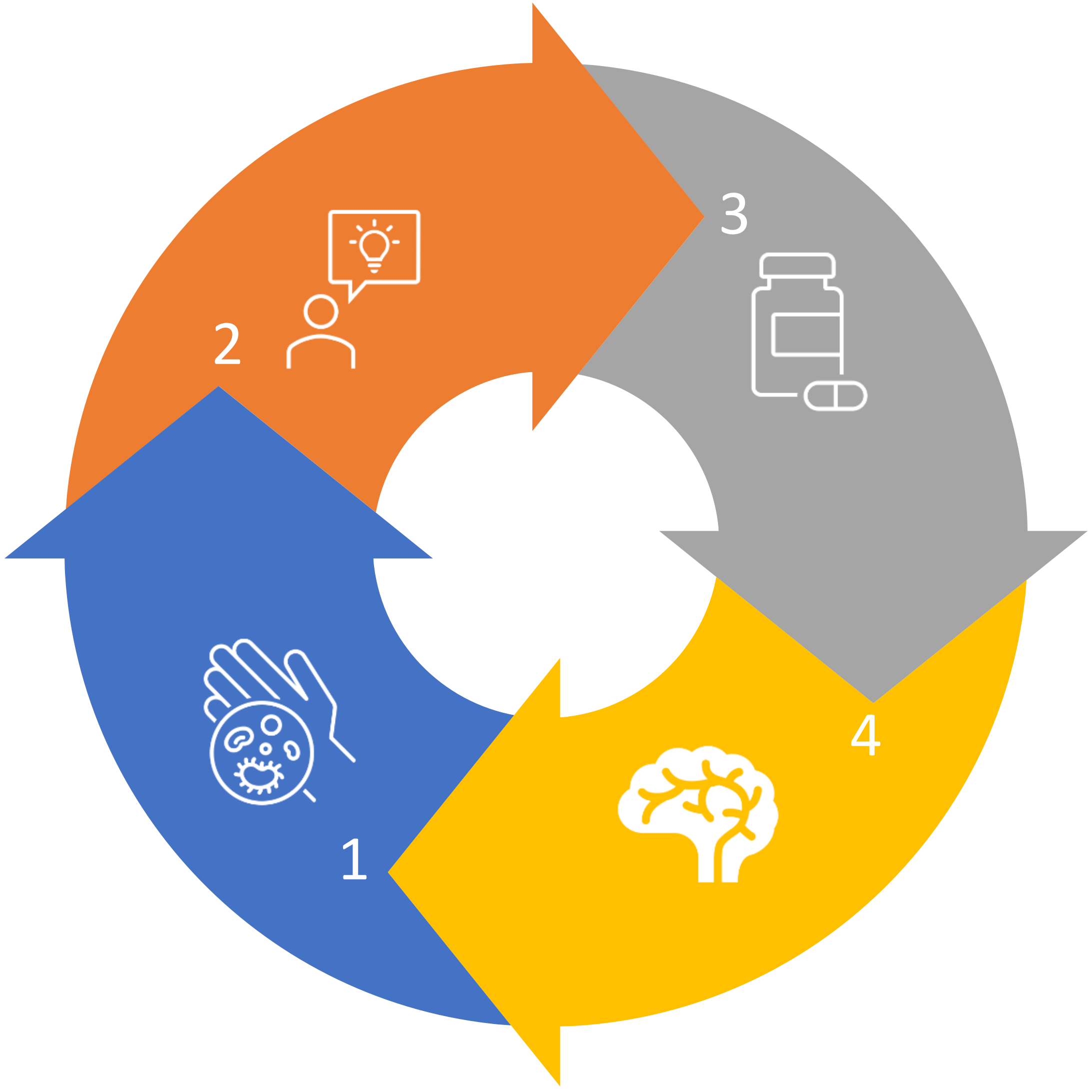

1 Common skin conditions and the knowledge gaps

- Dermatophytosis (*Daad; Daaj* were most common skin conditions
- Melasma, scabies, vitiligo, and acne were other common conditions
- Patients did not know the severity of these skin conditions, and often thought were caused by weather, sun exposure and sweating

2 Knowledge gap on the need for treatment

- Poor knowledge about the skin conditions, their cause and potentials of transmission
- Poor knowledge on how to deal with such skin conditions, and the treatment process
- Lack of knowledge on how these skin conditions could evolve, and complicate
- Perceived lack of severity of the skin conditions

3 Treatment seeking behaviour

- Patients first sought over the counter(OTC) medication including advice from family, friends, and neighbors
- Application of wide spectrum of home remedies
- OTC ointments often included steroids that suppressed the conditions for some time
- Inadequate recovery, or recurrence prompted them to seek treatment at the formal health services

4 Perceptions on the skin condition and treatment

- Patients perceived the cost of treatment at formal health services to be expensive
- Patients pronounced barriers to reaching formal health services (distance, the fact that they have to leave their work, travel arrangements)
- Stigma attached to skin conditions made some patients to conceal their condition deterring them to visit specialists.

S1

Click here to access/download

Supporting Information

Supporting Information File 1 COREQ_Checklist.pdf

S2

Click here to access/download
Supporting Information
Supporting Information File 2 Interview guide.docx

S3

Click here to access/download
Supporting Information
Supporting Information File 3 Inclusivity in Global Health.docx
