# Supplementary material for: Over the counter use of topical corticosteroid for skin conditions among patients before attending skin specialist clinic in Nepal: A qualitative study
Source: PLOS Glob Public Health. 2025 Jun 24;5(6):e0004812. doi: 10.1371/journal.pgph.0004812 (PMC12186941; doi:10.1371/journal.pgph.0004812)
Supplement: Attachment — Submitted filename: RESPONSE TO REVIEWERS COMMENTS.docx [file pgph.0004812.s006.docx]

13^th^ March 2025

Dear editor and the reviewers,

Thank you very much for your suggestion and opportunity to revise our work. We have utilized your suggestions to improve our manuscript. Please see below our responses to your specific suggestions with corresponding changes in the manuscript.

We look forward to your kind consideration.

On behalf of all the authors,

Yogesh Poudyal

PGPH-D-24-02509
Over the counter use of topical corticosteroid for skin conditions among patients before attending skin specialist clinic in Nepal: a qualitative study.
PLOS Global Public Health

Dear Dr. Poudyal,

Thank you for submitting your manuscript to PLOS Global Public Health. After careful consideration, we feel that it has merit but does not fully meet PLOS Global Public Health’s publication criteria as it currently stands. Therefore, we invite you to submit a revised version of the manuscript that addresses the points raised during the review process.

The manuscript has been evaluated by three reviewers, and their comments are available below.

The reviewers have raised a number of concerns that need attention. In particular, they request additional information on methodological aspects of the study, and further discussion.

Could you please revise the manuscript to carefully address the concerns raised?

Please submit your revised manuscript by Apr 19 2025 11:59PM. If you will need more time than this to complete your revisions, please reply to this message or contact the journal office at globalpubhealth@plos.org. When you're ready to submit your revision, log on to https://www.editorialmanager.com/pgph/ and select the 'Submissions Needing Revision' folder to locate your manuscript file.

Please include the following items when submitting your revised manuscript:

- A rebuttal letter that responds to each point raised by the editor and reviewer(s). You should upload this letter as a separate file labeled 'Response to Reviewers'.
- A marked-up copy of your manuscript that highlights changes made to the original version. You should upload this as a separate file labeled 'Revised Manuscript with Track Changes'.
- An unmarked version of your revised paper without tracked changes. You should upload this as a separate file labeled 'Manuscript'.

Guidelines for resubmitting your figure files are available below the reviewer comments at the end of this letter.

We look forward to receiving your revised manuscript.

Kind regards,

Helen Howard
Staff Editor
PLOS Global Public Health

Journal Requirements:

1. Please provide separate figure files in .tif or .eps format.

For more information about figure files please see our guidelines:
https://journals.plos.org/globalpublichealth/s/figures
https://journals.plos.org/globalpublichealth/s/figures#loc-file-requirements

Additional Editor Comments (if provided):


[Note: HTML markup is below. Please do not edit.]

Reviewers' comments:

Reviewer's Responses to Questions

**Comments to the Author**

1. Does this manuscript meet PLOS Global Public Health’s [publication criteria](https://journals.plos.org/globalpublichealth/s/criteria-for-publication)? Is the manuscript technically sound, and do the data support the conclusions? The manuscript must describe methodologically and ethically rigorous research with conclusions that are appropriately drawn based on the data presented.

Reviewer #1: Yes

Reviewer #2: Yes

Reviewer #3: Yes

2. Has the statistical analysis been performed appropriately and rigorously?

Reviewer #1: Yes

Reviewer #2: N/A

Reviewer #3: N/A

3. Have the authors made all data underlying the findings in their manuscript fully available (please refer to the Data Availability Statement at the start of the manuscript PDF file)?

The [PLOS Data policy](https://journals.plos.org/globalpublichealth/s/data-availability) requires authors to make all data underlying the findings described in their manuscript fully available without restriction, with rare exception. The data should be provided as part of the manuscript or its supporting information, or deposited to a public repository. For example, in addition to summary statistics, the data points behind means, medians and variance measures should be available. If there are restrictions on publicly sharing data—e.g. participant privacy or use of data from a third party—those must be specified.

Reviewer #1: Yes

Reviewer #2: Yes

Reviewer #3: Yes

4. Is the manuscript presented in an intelligible fashion and written in standard English?

PLOS Global Public Health does not copyedit accepted manuscripts, so the language in submitted articles must be clear, correct, and unambiguous. Any typographical or grammatical errors should be corrected at revision, so please note any specific errors here.

Reviewer #1: Yes

Reviewer #2: Yes

Reviewer #3: Yes

5. Review Comments to the Author

Please use the space provided to explain your answers to the questions above. You may also include additional comments for the author, including concerns about dual publication, research ethics, or publication ethics. (Please upload your review as an attachment if it exceeds 20,000 characters)

**Reviewer #1**:

1. **Methods: Study period (Sept 2021 to March 2022: Was there any rationale for the period of the study, as around 40 skin specific patients were present in the clinic per day and your sample size (N=31)?**

**AUTHORS:** Thank you for the question. As a dermatologist taking care of 40 patients in a clinic, I had to prioritize the clinical care first and only asked if anyone was willing to wait for the interview towards the end of the clinic hour. That was a big ask to patients, especially when their priority was to get treatment and travel back to their villages. As reported in the manuscript, most patients only came to the urban centre specialist clinic when they have exhausted the treatment in their village or nearby hospitals. This meant that they travelled from far, and the fact that they were only out-patients, they were in rush to return back. In addition, sometimes, my own workload would exhaust me to interview a patient at the end of the clinic. Given these circumstances, I chose to interview patients when number of patients were less in the clinic in a particular day, and after the end of the clinic hours. In addition, since we wanted specific cases of how they have navigated the health services for the particular skin condition (purposive selection), this also made us to recruit patients carefully so that they are the best informants to respond to our research question. In this way, it took me longer than expected time to complete 31 patients.

We have added a statement where we have mentioned the study duration to demonstrate how participants were selected and reads as follows:

Participants were also selected based on their logistical feasibility to sit for interviews after the clinical services.

**2. Clearly specify the inclusion and exclusion criteria of the study subjects.**

**AUTHORS:** Thank you for the suggestion. We have revised the last paragraph under the section ‘selection of participants’ and reads as follows:

YP is a dermatologist (MBBS, MD) with more than 10 years of dermatological practice in the region. The interviews were conducted by YP with participants meeting following inclusion criteria: any participant aged 18 years or older who had experienced a long-standing skin condition that failed to recover after home-based treatments or treatment elsewhere. Participants were excluded if they were unwilling to allocate time for the interviews, had long-standing skin conditions but were seeking treatment for the first time at the clinic, were unsure about past treatments received, or had co-morbid conditions requiring concomitant treatments. In addition, individuals who were not physically or mentally fit, or unwilling to provide written informed consent were respected and excluded from the study. Confidentiality of the data was maintained throughout the research process.

**3. Respondent Selection (L10): Was the dispensers (pharmacist) attitude towards TCs, OTC dispensing verified?**

**AUTHORS:** Thank you for the question. Since this study only interviewed patients attending the skin clinic, we did not interview or seek verification from pharmacists or dispensers. Nonetheless, when participants in Nepal responded with 'pharmacist,' they may have used the term loosely to refer to anyone working in a pharmacy or drug shop as a dispenser. We have added dispenser alongside the word ‘pharmacist’ in the result section to demonstrate the use of term.

**4. Results (Broad Themes & Consolidation of Themes):
a + b = 1. Skin conditions and the knowledge gaps;
c = 2. Treatment seeking behavior
d, where dis you consolidate d (perception) as 'c ' corresponds to practice and not perception?**

**AUTHORS:** Thank you for the question. We consolidated themes and sub-themes reducing them into two major headings: ‘Skin conditions and the knowledge gaps’ and ‘Treatment seeking behavior’. As shown in figure 1, we combined a) common skin conditions and knowledge gaps and b) knowledge gap on the need for treatment under the heading ‘Skin conditions and the knowledge gaps.’ We combined a) treatment seeking behavior and b) perception on the skin condition and treatment. We agree that it does have practices blended in patients’ perception as well. Since we interviewed these patients, and it’s their perceived responses related to the practices including treatment seeking behavior, therefore we chose it under the heading of ‘perception’.

**5. Table 1: (Age) You have conducted a face to face interview with socio-demographic details. Mention the exact age and NOT in range.. Example 1) 48 years, 2) 26 years etc.**

**AUTHORS:** We appreciate your comment, nonetheless, since this is a qualitative study, we thought to add age range rather than exact age to ensure that they are potentially identifiable by their quotes on the manuscript. Instead, the age range were utilized following the presentation style in qualitative articles where exact age is deliberately concealed for the confidentiality and complete anonymization.

**6. Discussion: Was there any discussion done with the pharmacists (drug store or pharmacies} to understand the trend for self medication. Did'nt the pharmacist refer them to specialist clinics? What is the basis of the statement?**

**AUTHORS:** Thank you for the question. Apart from the observation notes that the first author (YP) collected, we did not engage with the dispensers or the drug shop owners. We entirely relied on the patients’ responses, observation notes and authors’ experiential knowledge. Therefore, these statements are based on the respondents’ (patients/careseekers) experience of interacting with the drug shops. These are corroborated by several previous reports including the [recent one](https://gh.bmj.com/content/6/5/e005829).

**7. P16, para 2: "These medical shops were preferred because they involved minimal direct, indirect, and opportunity costs, making them an attractive option for many individuals." - Was there any discussion done with the pharmacists (drug store or pharmacies} to understand the trend for self medication. Did'nt the pharmacist refer them to specialist clinics. What is the basis of the statement?**

**AUTHORS:** Thank you for the question. As above, in this study, we did not interview the drug dispensers or drug shop owners, but it has been quite established by previous studies that drug stores tend to provide OTC medications to most of the out-patients unless, they were severe enough requiring treatment by the specialists. Yes, they do refer in case of severity, but most of the non-severe cases are offered OTC medications.

<https://www.cabidigitallibrary.org/doi/pdf/10.5555/20241604383>

<https://kathmandupost.com/health/2024/01/27/report-reveals-65-percent-of-patients-in-nepal-given-antibiotics-without-prescription>

<https://pmc.ncbi.nlm.nih.gov/articles/PMC9461566/>

<https://www.nature.com/articles/s41598-021-90812-4>

<https://gh.bmj.com/content/6/5/e005829>

**8. Reference: Please check the journal referencing guidelines and edit all the references accordingly.**

**AUTHORS:** Thank you for the suggestion. We have revised the referencing style according to the journal requirement.

**Reviewer #2: PGPH-D-24-02509**
**Review steroid
 Comments: Abstract: Conclusion: suggest switching the word “insurmountable” to something more optimistic and achievable eg difficult or tough as want to indicate potential solution not just a problem that cannot be fixed.**

**AUTHORS:** Thank you for the suggestion. We have revised the conclusion and replaced the word ‘insurmountable’. The revised conclusion reads as follows:

## Conclusions

OTC use of topical medications poses significant challenges, often leading to complicated skin cases that present late at skin-specialist clinics. A multi-pronged public and community engagement approach is critical to curb the treatment seeking dynamics among patients developing skin conditions.

**Introduction**
**Suggest emphasizing that dermatophytosis as a superficial fungal infection is in the top 4 of all disease burdens in GBD study (Vos T., Abajobir A.A., Abate K.H. Global, regional, and national incidence, prevalence, and years lived with disability for 328 diseases and injuries for 195 countries, 1990-2016: a systematic analysis for the Global Burden of Disease Study 2016. Lancet. 2017;390(10100):1211–1259. doi: 10.1016/S0140-6736(17)32154-2) This is the hook to bring in the general reader.**

**AUTHORS:** Thank you for the suggestion. We have added the reference and added a statement which reads as follows:

In Nepal, many topical preparations are sold in pharmacies as OTC medications (the price could range from 0.5 USD to 4 USD), i.e. these can be given to patients without doctor’s prescription and there are no definite guidelines for OTC use and prescription medications [14]. Dermatophytosis is a common disorder of skin also commonly known as ringworm and is a fungal infection of the skin, hair, or nails [15]. Dermatophytosis, a common superficial fungal infection, ranked among the top four disease burdens globally in the 2016 Global Burden of Disease study [16]. Among skin conditions, dermatophytosis has long been one of the most common skin infections, the significant shift in clinical profile, with rise in chronic, recurrent , or treatment resistant cases is concerning [17]. This resistance poses a major challenge to achieving a complete cure. Easy availability and widespread uses of irrational combination cream containing potent steroid, anti-fungal and anti-bacterial are deemed to be one of the most significant reasons for alarming outbreak of Tinea and recalcitrant type of dermatophytosis [18, 19]. Moreover in Nepal, dermatology clinics frequently deal with steroid induced and steroid aggravated dermatosis [20]. Managing these complications is often more difficult and distressing for patients than treating the original condition for which steroids were inappropriately used [21]. In more than 93% of the skin cases, TC is used unnecessarily for an extended duration beyond requirement, of inappropriate potency or administered without a thorough diagnosis of underlying condition [11].

**Also suggest including a brief explanation of the access to dermatology care options in Nepal, Do all patient have to pay is there free care available via a public system? You discuss this a bit in the discussion section but I think it needs summarising in the introduction and study context.
Would be good to highlight the global context of this issue possibly referencing the ILDS/AAD platform "Dermatology Covid-19, Monkeypox, and Emerging Infections Registry"** [**https://redcap.partners.org/redcap/surveys/index.php?s=YJWAJCX7TY**](https://redcap.partners.org/redcap/surveys/index.php?s=YJWAJCX7TY)

**AUTHORS:** Thank you for the suggestion. Adding access to dermatology care will provide background for steroid misuse and will also help reader to understand the context. We have also highlighted the global context of this issue. We have revised the paragraphs incorporating your suggestions as below:

In Nepal, many topical preparations are sold in pharmacies as OTC medications (the price could range from 0.5 USD to 4 USD), i.e. these can be given to patients without doctor’s prescription and there are no definite guidelines for OTC use and prescription medications [14]. Dermatophytosis is a common disorder of skin also commonly known as ringworm and is a fungal infection of the skin, hair, or nails [15]. Dermatophytosis, a common superficial fungal infection, ranked among the top four disease burdens globally in the 2016 Global Burden of Disease study [16]. Among skin conditions, dermatophytosis has long been one of the most common skin infections, the significant shift in clinical profile, with rise in chronic, recurrent , or treatment resistant cases is concerning [17]. The American Academy of Dermatology (AAD) and the International League of Dermatological Societies (ILDS) have been gathering data on treatment-resistant tinea (dermatophytosis) through the “AAD/ILDS Dermatology COVID-19, Monkeypox, and Emerging Infections Registry” [18]. The OTC use of topical steroids has been identified as a contributing factor to treatment-resistant tinea [19]. This resistance poses a major challenge to achieving a complete cure. Easy availability and widespread uses of irrational combination cream containing potent steroid, anti-fungal and anti-bacterial are deemed to be one of the most significant reasons for alarming outbreak of Tinea and recalcitrant type of dermatophytosis [19, 20].

In Nepal, most of the population resides in remote areas where healthcare is provided by health assistants in health posts and general practitioners in primary health care centers and district hospitals [21]. While the Ministry of Health offers basic healthcare services, the services in rural areas are largely limited despite being mostly free [21, 22]. Specialist care, such as dermatology, is limited to hospitals in major cities or medical colleges [22]. Due to inadequate training, medical officers and health workers often lack the expertise to manage common skin diseases, leaving much of Nepal’s population without access to dermatological care [23, 24]. Moreover in Nepal, dermatology clinics frequently deal with steroid induced and steroid aggravated dermatosis [25, 26]. Managing these complications is often more difficult and distressing for patients than treating the original condition for which steroids were inappropriately used [27]. In more than 93% of the skin cases, TC is used unnecessarily for an extended duration beyond requirement, of inappropriate potency or administered without a thorough diagnosis of underlying condition [11].

**Method
Study setting
You indicate the study was done in a clinic which I assume is private although not clear. How much do the patient pay to access? Where else could they go? Is there dermatology services available in the local tertiary hospital you mention,**

**AUTHORS:** Thank you for suggestions. We have reivsed the paragraph under the ‘study setting’ and reads as follows:

## Study settings

This study was conducted at Sarnath Skin Centre, a private dermatology clinic in the urban center of Bhairahawa. Established in 2017, the clinic serves approximately 40 patients daily, primarily those with skin-related conditions. Consultation fees at such clinics in the area typically range from USD 5 to 7.

Bhairahawa, officially known as Siddharthanagar, is a municipality and the administrative center of Rupandehi District in Lumbini Province, Nepal. Located on the southern plains near the Indian border, the city has a population of approximately 75,000 [30]. The city has a tertiary hospital hosted by Universal College of Medical Sciences with specialist clinics including dermatology. There are private hospitals and clinics where patients also seek specialist care.

Bhairahawa as an administrative and urban center of the district receives patients from the settlements around the urban center including patients from northern mountains who visit for specialist clinic care. Most of the patients attending the specialist clinic came from outskirts of the urban Bhairahawa and were mostly working in the farmlands, particularly rice fields.

**You indicate you recruit over 6 months when the clinic would have seen about 5000 patients and yet you interview/include just 31 patients (less than 1%) – Given the premise of the study that steroid OTC is a common problem in your setting, can you indicate how you selected the cases? Presumably you did not include all patients who admitted to using OTC steroid as this must be higher than <1%. I think it would help the manuscript to understand more about the case selection process and what about those who you identified but did not take part would strengthen the validity of the conclusions you then draw**

**AUTHORS:** Thank you for the comment, it was also raised by reviewer #1. As a dermatologist taking care of 40 patients in a clinic, I had to prioritize the clinical care first and only asked if anyone was willing to wait for the interview towards the end of the clinic hour. That was a big ask to patients, especially when their priority was to get treatment and travel back to their villages. As reported in the manuscript, most patients only came to the urban centre specialist clinic when they have exhausted the treatment in their village or nearby hospitals. This meant that they travelled from far, and the fact that they were only out-patients, they were in rush to return back. In addition, sometimes, my own workload would exhaust me to interview a patient at the end of the clinic. Given these circumstances, I chose to interview patients when number of patients were less in the clinic in a particular day, and after the end of the clinic hours. In addition, since we wanted specific cases of how they have navigated the health services for the particular skin condition (purposive selection), this also made us to recruit patients carefully so that they are the best informants to respond to our research question. In this way, it took me longer than expected time to complete 31 patients.

We have added a statement under the heading ‘selection of study participants’ on how participants were selected and reads as follows:

Participants were also selected based on their logistical feasibility to sit for interviews after the clinical services. **In terms of conclusions
Referencing in the International League of Dermatologists Position Statement on the safe and appropriate use of topical steroids might strengthen your message as will calling for an increase in including access to skin health as a global public health priority and essential for universal health coverage alluding to the recently proposed and put forward as an agenda item for World Health Assembly in May 2025
See concept note @** [**https://globalskin.org/doclink/skin-diseases-as-a-global-public-health-priority/eyJ0eXAiOiJKV1QiLCJhbGciOiJIUzI1NiJ9.eyJzdWIiOiJza2luLWRpc2Vhc2VzLWFzLWEtZ2xvYmFsLXB1YmxpYy1oZWFsdGgtcHJpb3JpdHkiLCJpYXQiOjE3MTY0OTc4NzksImV4cCI6MTcxNjU4NDI3OX0.1VKfoy3Nd_ZJMr-JI0uCV1cSSI41uCjrDjSrbdMmNk0#:~:text=NTD%202021%20%2D2030%20roadmap%20and,of%20resources%2C%20policy%20and%20advocacy.&text=previous%20unmet%20need%2C%20expand%20access,target%20to%20deliver%20on%20UHC**](https://globalskin.org/doclink/skin-diseases-as-a-global-public-health-priority/eyJ0eXAiOiJKV1QiLCJhbGciOiJIUzI1NiJ9.eyJzdWIiOiJza2luLWRpc2Vhc2VzLWFzLWEtZ2xvYmFsLXB1YmxpYy1oZWFsdGgtcHJpb3JpdHkiLCJpYXQiOjE3MTY0OTc4NzksImV4cCI6MTcxNjU4NDI3OX0.1VKfoy3Nd_ZJMr-JI0uCV1cSSI41uCjrDjSrbdMmNk0#:~:text=NTD%202021%20%2D2030%20roadmap%20and,of%20resources%2C%20policy%20and%20advocacy.&text=previous%20unmet%20need%2C%20expand%20access,target%20to%20deliver%20on%20UHC)**.**

**AUTHORS:** Thank you for this useful suggestion. We have revised the paragraph under ‘implications’ and reads as follows:

The widespread use of topical corticosteroid, including advertisements over media on beauty products require immediate scrutiny so that these products are regulated. The International League of Dermatological Societies (ILDS), an umbrella organization for professional dermatological societies, has released a position paper with two key recommendations [51] that align with this study’s findings:

- Moderate to strong topical corticosteroids, whether alone or combined with other active compounds like antimicrobials, should only be used when prescribed by a properly trained health professional.
- In countries where unregulated use is common, clear guidelines should be in place to protect patients and the public from overuse or misuse.

The ILDS recommendations, along with the World Health Assembly's recognition of skin health as a global public health priority for universal health coverage in May 2024, highlight the urgent need to strengthen primary health care in low- and middle-income countries [52].

**Reviewer #3:** **This article is a very important in the current scenario of topical steroid abuse in our country. It is good to see article related to topical steroid misuse being published from public health point but it would have been better if the authors could also do analysis of the responses.**

**AUTHORS:** Thank you for your evaluation and feedback. As this is a qualitative analysis, we followed the standard thematic analysis approach for the textual data, which is why we used qualitative methods. In the future, incorporating quantitative data collection and analysis could be a valuable direction. We have included this as a potential avenue for future research based on the findings of this study. We have added a statement within ‘implications’ section to reflect on this and reads as follows:

Future studies could integrate quantitative data collection and analysis to complement the qualitative findings, including exploring the magnitude of OTC steroid use for skin conditions.

6. PLOS authors have the option to publish the peer review history of their article ([what does this mean?](https://journals.plos.org/globalpublichealth/s/editorial-and-peer-review-process#loc-peer-review-history)). If published, this will include your full peer review and any attached files.


**Do you want your identity to be public for this peer review?** If you choose “no”, your identity will remain anonymous but your review may still be made public.


For information about this choice, including consent withdrawal, please see our [Privacy Policy](https://www.plos.org/privacy-policy).

Reviewer #1: **Yes:** Abdul Nazer Ali

Reviewer #2: **Yes:** Dr Lucinda Claire Fuller

Reviewer #3: No
