# Supplementary material for: Over the counter use of topical corticosteroid for skin conditions among patients before attending skin specialist clinic in Nepal: A qualitative study
Source: PLOS Glob Public Health. 2025 Jun 24;5(6):e0004812. doi: 10.1371/journal.pgph.0004812 (PMC12186941; doi:10.1371/journal.pgph.0004812)
Supplement: Attachment — Submitted filename: RESPONSE_TO_REVIEWERS_COMMENTS_auresp_2.docx [file pgph.0004812.s008.docx]

11^th^ April 2025

Dear editor and the reviewers,

Thank you very much for your suggestion and opportunity to revise our work. We have utilized your suggestions to improve our manuscript. Please see below our responses to your specific suggestions with corresponding changes in the manuscript.

We look forward to your kind consideration.

On behalf of all the authors,

Yogesh Poudyal

**Reviewer #1**: **No comments raised in a previous round (15. 11 2025) of review has been addresses nor justified. It is just the same manuscript sent wasting the reviewers time. However, Having reviewed again, I register the following comments,**

**AUTHORS:** Apologies, there must have been misunderstanding, mis-attachment or something. We did attend all the comments provided to us in the previous round and revised as sincerely as we could. We are grateful for your additional comments in this round, and we have revised based on your suggestions. Below we attend to each of the comments.

**The article addresses an important and under-researched topic: the over-the-counter (OTC) use of topical corticosteroids (TCS) in Nepal, particularly among patients before they seek specialized dermatological care. The study employs a qualitative approach, which is appropriate for exploring the motivations, perceptions, and experiences of patients regarding self-medication with TCS. The findings have significant implications for public health, dermatological practice, and policy-making in low-resource settings like Nepal. However, the manuscript requires revisions to strengthen its methodological rigor, clarity, and overall impact.**

**AUTHORS:** Thank you for your evaluation.  **Areas for Improvement:

1. Methodological Clarity:
- The sampling strategy is not clearly described. How were participants selected? What criteria were applied in purposive sampling?**

**AUTHORS:** Thank you for your question. We notice that you had this question/comment in the previous round of review, and we responded to this question and made changes in the manuscript. We have revised the last paragraph under the section ‘selection of participants’ and reads as follows:

YP is a dermatologist (MBBS, MD) with more than 10 years of dermatological practice in the region. The interviews were conducted by YP with participants meeting following inclusion criteria: any participant aged 18 years or older who had experienced a long-standing skin condition that failed to recover after home-based treatments or treatment elsewhere. Participants were excluded if they were unwilling to allocate time for the interviews, had long-standing skin conditions but were seeking treatment for the first time at the clinic, were unsure about past treatments received, or had co-morbid conditions requiring concomitant treatments. In addition, individuals who were not physically or mentally fit, or unwilling to provide written informed consent were respected and excluded from the study. Confidentiality of the data was maintained throughout the research process.

**- The interview guide should be included as a supplementary file or described in detail to ensure transparency and reproducibility.**

**AUTHORS:** Thank you for your suggestion. We have provided interview guide as a supplementary file 2 (S2 File) and have cited this within the manuscript under the section ‘Data collection guide’.

**- The process of data saturation is mentioned but not adequately explained. How was saturation determined?**

**AUTHORS:** Thank you for your suggestion. We have revised the statement under the section ‘Selection of respondents’ to make it clear and read as follows:

All respondents were selected purposively in this study, and the total number of participants was determined based on data saturation [1]. The data saturation in this study was considered achieved when additional interviews began to yield repeated themes, insights, nuances, and understandings of the phenomenon [2].

**2. Data Analysis:
- The article lacks detail on the qualitative data analysis process. Was a specific framework (e.g., thematic analysis, grounded theory) used? How were themes identified and validated? Including a coding tree or thematic framework as a supplementary file would enhance transparency.**

**AUTHORS:** Thank you for your suggestion. We utilized reiterative process of thematic analysis (six phases) as described by Braun and Clarke 2006. Based on these six phases of thematic analysis, themes were refined and supplemented by excerpts supporting them. We have revised the section ‘data analysis’ and reads as follows:

## Data Analysis

Participants' personal information was anonymized to protect their identity. All transcribed data were stored in Microsoft Word and then analyzed by two coders (NA and AR) using NVivo software. Six phases of thematic analysis outlined by Braun and Clarke [3] were utilized that included 1. Familiarizing with the data (transcribing data, reading and rereading the data, noting down initial ideas); 2. Generating initial codes (coding interesting features of the data systematically across the entire data set, collating data relevant to each code); 3. Searching for themes (collating codes into potential themes, gathering all data relevant to each potential theme); 4. Reviewing themes (checking if the themes work in relation to the coded extracts and the entire data set, generating a thematic map); 5. Defining and naming themes (ongoing analysis to refine the specifics of each theme, and the overall story the analysis); and 6. Producing the report (selection of vivid, compelling extract examples, final analysis of selected extracts, relating back of the analysis to the research question and literature, producing a scholarly report of the analysis) [3, 4].

The thematic analysis in this study entailed a mix of deductive and inductive approaches to coding of data and was critically reviewed by BA, YP and BJ. The major themes and their corresponding sub-themes, derived from coding and subsequent thematic synthesis, are shown in Figure 1. The analyzed data were thoroughly cross-checked for any mis-association and misinterpretations. By focusing on the subjective experiences of the participants, the research sought to uncover deeper meanings and insights into their personal events.

**- The role of the researchers in the analysis process (e.g., reflexivity, potential biases) is not discussed. This is important for establishing the credibility of the findings.**

**AUTHORS:** Thank you for your suggestion. We have added a section on ‘reflexivity’ and reads as follows:

## Reflexivity

YP, the principal investigator and treating dermatologist, conducted the interviews with patients (participants). As a dermatologist, YP’s interactions with patients may have been influenced by epistemological and power dynamics, potentially introducing social desirability bias in the responses.

NA and AR, both Nepalese public health practitioners, independently coded the data and generated the initial codes and themes. YP and BA participated regularly in the coding and thematic synthesis processes. BA is a social scientist and Nepali medical practitioner with prior research experience on the over-the-counter use of antimicrobials in Nepal; he provided input on coding and the final thematic synthesis.

MG, SA, SR, BJ, and SP are medical doctors with extensive experience in Nepal’s healthcare landscape. PYC is a professor of ethics with significant collaborative research experience on antimicrobial resistance in South and Southeast Asia.

**3. Findings and Interpretation:
- The results section is descriptive but could benefit from deeper interpretation. For example, what do the findings imply about the cultural or socio-economic factors driving OTC use of TCS in Nepal?**

**AUTHORS:** We appreciate your suggestion. The entire results section presents an interpretative analysis that integrates the findings from participants with the local social and cultural context of Nepal. The lived experiences of the authors—as physicians, public health practitioners, and social scientists—enabled us to interpret the data in ways that align with this context. We have made conscious efforts to strike a balance, avoiding both over-interpretation and misinterpretation by staying close to the descriptive findings. In the discussion section, we have also included a dedicated segment on the implications of our findings, outlining recommended steps for policymakers and stakeholders.

**- The article does not sufficiently contextualize the findings within the broader literature on OTC medication use in LMICs. How do the results compare with similar studies in other regions?**

**AUTHORS:** We appreciate your question. We have revised the second paragraph of the discussion section to better reflect literature from the region. While the previous version primarily compared our findings with studies from India—given its close approximation to Nepal’s medical, social, and cultural context—the revised paragraph takes a broader perspective. It now discusses how over-the-counter (OTC) medicine use occurs both within and beyond the region, particularly in low- and middle-income countries (LMICs). The revised paragraph reads as follows:

Most case of steroid misuse noted in this study was dermatophytosis. Tinea incognito and acne were most frequent complications noted in patients misusing steroid in India [5]. A study by Molly et al [6] showed 58% of the participant perceived their skin condition to be allergic to food when in fact 70.1% were Tinea. A number of studies from India, a regional neighbor, report high use of topical corticosteroids among dermatology patients and continue to highlight this as a major public health concern [7, 8]. A recent review also demonstrated widespread use of topical corticosteroids (TCs) within the Indian subcontinent, identifying it as a significant issue encountered in dermatology clinics [9]. The use of topical corticosteroids beyond the Indian subcontinent—such as in Southeast Asia—has similarly been reported as a major public health concern [10]. The over-the-counter (OTC) use of TCs reflects broader trends seen with other medications both within and outside the region, particularly in low- and middle-income countries (LMICs) [11]. Close to our study site, a study conducted in Bhairahawa showed that those who consulted a dermatologist were less likely to misuse steroids [12]. Our study also showed participants had poor knowledge of skin disease. This might have paved the way for steroid misuse.

**4. Ethical Considerations:
- Ethical approval and informed consent are mentioned, but more details are needed. Were participants informed about the potential risks of discussing sensitive topics? How was confidentiality maintained?**

**AUTHORS:** We appreciate your question. We have revised the paragraph under the section ‘selection of respondents’ and reads as follows:

………………. Participants were first verbally asked if they were interested in participating in the study during their clinical examination. Each prospective participant was briefed on the study, its procedures, and its implications, as outlined in the informed consent form. It was explained that their anonymized perspectives would contribute to the study findings, and the only potential risk—that they would need to share details about their skin conditions and the treatments they had sought—was clearly communicated. Once they verbally agreed, a written informed consent was obtained and were interviewed in the separate room within the clinic premise. Confidentiality was maintained by conducting interviews in private within the clinic, securely storing data on the first author’s password-protected laptop, sharing data only among co-authors for analysis and interpretation, anonymizing the data when reporting, and removing identifiers wherever possible.

**5. Writing and Structure:
- The introduction could be more concise and focused. The background information on TCS and their misuse is relevant but could be streamlined to better highlight the research gap.**

**AUTHORS:** Thank you for the suggestion. We have revised the introduction/background section by consolidating further to streamline the essential insights and highlight the research gap.

**- The discussion section should be expanded to include recommendations for policy and practice. For example, how can healthcare providers and policymakers address the issue of OTC TCS use in Nepal?**

**AUTHORS:** Thank you for the suggestion. This may have been an oversight. However, in our most recent revision, we added a dedicated section within the discussion—titled ‘Implications’—where we outline recommendations for both policy and practice. We have further revised the section to include additional implications discussed among the authors.

**6. Limitations:
- The limitations of the study are not adequately addressed. For instance, the generalizability of the findings to other regions or populations is limited due to the qualitative design and specific context of Nepal. This should be explicitly acknowledged.**

**AUTHORS:** Thank you for the suggestion. We have added the limitation as you suggested within the section ‘strengths and limitations of study’ that reads as follows:

The use of a qualitative method with a specific cohort of participants from a specialist clinic limits the generalizability of the findings to broader social and cultural contexts.

**Reviewer #2**: Thanks for this opportunity to review this revised manuscript on an important topic.
I hope the comments are helpful

**Abstract Introduction
Suggest you change the word “use” to “access” or “availability” The current sentence doesn’t make sense.**

**AUTHORS:** Thank you for the suggestion. Revised as suggested.

**Introduction:
The reference supporting your opening statement that steroids have emerged as the primary choice for dermatologists in the outpatient settings is a review of the use of steroids and does not include source data to support this claim. The statement is made in the reference with no supporting source.

Suggest you rephrase to say TCs are widely prescribed by the dermatologist…….etc
Das A, Panda S. Use of topical corticosteroids in dermatology: an evidence-based approach. Indian journal of dermatology. 2017;62(3):237-50**

**AUTHORS:** Thank you for the suggestion. Revised as suggested.

**Might you prepare a summary of the participants in terms of male/female, and number in each age range, with the actual age of the participants on your full table of demographics.**

**AUTHORS:** Thank you for the suggestion. In line with qualitative methodological traditions, we aimed to minimize the disclosure of socio-demographic details that could serve as identifiers. In addition, in accordance with the principle of confidentiality and adequate anonymization, disclosing actual ages could potentially compromise participant anonymity. Therefore, we kindly request your consideration in allowing the presentation of age ranges, as shown in Table 1.

**References**

1.	Saunders B, Sim J, Kingstone T, Baker S, Waterfield J, Bartlam B, et al. Saturation in qualitative research: exploring its conceptualization and operationalization. Qual Quant. 2018;52(4):1893-907. Epub 2018/06/26. doi: 10.1007/s11135-017-0574-8. PubMed PMID: 29937585; PubMed Central PMCID: PMCPMC5993836.

2.	Hennink M, Kaiser BN. Sample sizes for saturation in qualitative research: A systematic review of empirical tests. Social science & medicine. 2022;292:114523.

3.	Braun V, Clarke V. Using thematic analysis in psychology. Qualitative research in psychology. 2006;3(2):77-101.

4.	Vaismoradi M, Turunen H, Bondas T. Content analysis and thematic analysis: Implications for conducting a qualitative descriptive study. Nursing & health sciences. 2013;15(3):398-405.

5.	Meena S, Gupta LK, Khare AK, Balai M, Mittal A, Mehta S, Bhatri G. Topical corticosteroids abuse: A clinical study of cutaneous adverse effects. Indian journal of dermatology. 2017;62(6):675.

6.	Thomas M, Wong CC, Anderson P, Grills N. Magnitude, characteristics and consequences of topical steroid misuse in rural North India: an observational study among dermatology outpatients. BMJ open. 2020;10(5):e032829.

7.	Kumar S, Goyal A, Gupta YK. Abuse of topical corticosteroids in India: Concerns and the way forward. Journal of Pharmacology and Pharmacotherapeutics. 2016;7(1):1-5.

8.	Nagesh T, Akhilesh A. Topical steroid awareness and abuse: A prospective study among dermatology outpatients. Indian journal of dermatology. 2016;61(6):618-21.

9.	Satheesh G, Puthean S, David E. The unattended crisis of topical steroid misuse: A review. SM J Pharmac Ther. 2018;4(1):1020.

10.	Handog EB, Enriquez-Macarayo MJ. Topical corticosteroid abuse: Southeast Asia perspective. A Treatise on Topical Corticosteroids in Dermatology: Use, Misuse and Abuse: Springer; 2017. p. 209-22.

11.	Mao W, Vu H, Xie Z, Chen W, Tang S. Systematic review on irrational use of medicines in China and Vietnam. PloS one. 2015;10(3):e0117710.

12.	Poudyal Y, Joshi SD. Medication practice of patients with dermatophytosis. JNMA J Nepal Med Assoc. 2016;55(203):7-10.
