# Supplementary material for: Over the counter use of topical corticosteroid for skin conditions among patients before attending skin specialist clinic in Nepal: A qualitative study
Source: PLOS Glob Public Health. 2025 Jun 24;5(6):e0004812. doi: 10.1371/journal.pgph.0004812 (PMC12186941; doi:10.1371/journal.pgph.0004812)
Supplement: Attachment — Submitted filename: RESPONSE_TO_REVIEWERS_COMMENTS_auresp_3.docx [file pgph.0004812.s010.docx]

3rd May 2025

Dear Editor and Reviewers,

Thank you very much for your line-by-line review, suggestions on the revised manuscript, and the opportunity to revise our work. We have utilized your suggestions to improve the manuscript. Please find below our responses to your specific suggestions, along with the corresponding changes in the manuscript.

We look forward to your kind consideration.

On behalf of all the authors,
Yogesh Poudyal

PGPH-D-24-02509R2
Over the counter use of topical corticosteroid for skin conditions among patients before attending skin specialist clinic in Nepal: a qualitative study.
PLOS Global Public Health

Dear Dr. Poudyal,

Thank you for submitting your manuscript to PLOS Global Public Health. After careful consideration, we feel that it has merit but does not fully meet PLOS Global Public Health’s publication criteria as it currently stands. Therefore, we invite you to submit a revised version of the manuscript that addresses the points raised during the review process.

Your revised manuscript has been reassessed by reviewers 1 and 2. Reviewer 1 has some minor revisions that we would like you to address in your revised manuscript please, in addition to providing a point-by-point response. 

Please submit your revised manuscript by May 30 2025 11:59PM. If you will need more time than this to complete your revisions, please reply to this message or contact the journal office at globalpubhealth@plos.org. When you're ready to submit your revision, log on to https://www.editorialmanager.com/pgph/ and select the 'Submissions Needing Revision' folder to locate your manuscript file.

Please include the following items when submitting your revised manuscript:

- A rebuttal letter that responds to each point raised by the editor and reviewer(s). You should upload this letter as a separate file labeled 'Response to Reviewers'.
- A marked-up copy of your manuscript that highlights changes made to the original version. You should upload this as a separate file labeled 'Revised Manuscript with Track Changes'.
- An unmarked version of your revised paper without tracked changes. You should upload this as a separate file labeled 'Manuscript'.

Guidelines for resubmitting your figure files are available below the reviewer comments at the end of this letter.

We look forward to receiving your revised manuscript.

Kind regards,

Sarah Jose, Ph.D.
Staff Editor
PLOS Global Public Health

**Reviewer #1**:

1. **Review the manuscript for minor grammatical errors and typographical issues to improve clarity and flow.**

**AUTHORS:** Thank you for the suggestion on improving the readability. We have revised the manuscript to fix the grammar, typos, flow and clarity.

**2. If feasible, provide a coding framework or thematic map as supplementary material to enhance transparency of the analysis process.**

**AUTHORS:** Thank you for the suggestion. We have added a coding framework as a supplementary file 3 as suggested.

**3. Expand on systemic factors in discussion (e.g., pharmacy practices, regulation gaps, healthcare access) contributing to OTC corticosteroid use.**

**AUTHORS:** Thank you for the suggestion. We have added a discussion on systematic factors related to pharmacy practices, regulation gaps, and healthcare access contributing to OTC corticosteroid use. This has been added in fifth paragraph in Discussion section.

**4. Provide more concrete and actionable public health recommendations (e.g., public awareness campaigns, regulatory enforcement)**

**AUTHORS:** Thank you for the suggestion. The main objective of having the section ‘implications’ was to make recommendations that are practical. We have further revised the implication section to emphasize actionable public health recommendations.

**Reviewer #2**:

**The ILDS recommendations, along with the World Health Assembly's recognition of skin health as a global public health priority for universal health coverage in May 2024, highlight the urgent need to strengthen primary health care in low- and middle-income countries [59]

It is actually on the agenda for May 2025 (not 2024)**

**AUTHORS:** Thank you for the suggestion. We have revised the date as you suggested.
